# Synthesis of glycosylated β^3^-homo-threonine conjugates for mucin-like glycopeptide antigen analogues

**DOI:** 10.3762/bjoc.6.47

**Published:** 2010-05-12

**Authors:** Florian Karch, Anja Hoffmann-Röder

**Affiliations:** 1 Institut für Organische Chemie, Johannes Gutenberg-Universität Mainz, Duesbergweg 10–14, D-55128 Mainz, Germany, Phone: +49-6131-3922417, Fax: +49-6131-3923916

**Keywords:** glycopeptide, glycosylamino acids, β^3^-homo-threonine, MUC1 antigens, solid-phase synthesis

## Abstract

Glycopeptides from the mucin family decorated with tumour-associated carbohydrate antigens (TACA) have proven to be important target structures for the development of molecularly defined anti-cancer vaccines. The strategic incorporation of β-amino acid building blocks into such mucin-type sequences offers the potential to create pseudo-glycopeptide antigens with improved bioavailability for tumour immunotherapy. Towards this end, T_N_ and TF antigen conjugates *O*-glycosidically linked to Fmoc-β^3^-homo-threonine were prepared in good yield via Arndt–Eistert homologation of the corresponding glycosyl α-amino acid derivative. By incorporation of T_N_-Fmoc-β^3^hThr conjugate into the 20 amino acid tandem repeat sequence of MUC1 using sequential solid-phase glycopeptide synthesis, a first example of a mixed α/β-hybrid glycopeptide building block was obtained. The latter is of interest for the development of novel glycoconjugate mimics and model structures for anti-cancer vaccines with increased biological half-life.

## Introduction

Glycosylation is the predominant co- and post-translational modification in higher organisms responsible for tailoring and fine-tuning of the activity of proteins involved in fundamental biological recognition events of cell adhesion, cell differentiation and cell growth [1–3]. As a consequence, synthetic oligosaccharides and their conjugates are recognised as important tools for the expanding field of chemical biology [4]. Aberrant glycosylation of cell surface glycoproteins is associated with various pathological incidents, e.g., autoimmune and infectious diseases and cancer. In the latter case, unusual glycan structures composed of truncated *O*-linked oligosaccharides of carcinoma-derived mucin glycoproteins can be used as markers of the tumourigenic process and as target structures for cancer immunotherapy [5]. Over the last years, mucin-type glycopeptides decorated with tumour-associated carbohydrate antigens (TACA) have been shown to trigger strong humoral immunity within molecularly defined vaccine prototypes [6–10]. However, the limited metabolic stability of the glycopeptide conjugates represents a major obstacle for the development of efficient carbohydrate-based vaccines. Various strategies towards the incorporation of non-natural hydrolysis resistant carbohydrate analogues into vaccine constructs have been pursued. For instance, stable TACA mimics comprising C-glycosides [11–14], S-glycosides [15–19] and deoxyfluoro sugars [20] have been used to circumvent hydrolytic degradation by endogenous glycosidases.

In principle, antigenicity of the artificial TACA derivatives should be enhanced by minor structural modifications assuming that the conformations remain similar to those of the natural antigens. In this respect, hybrid peptides in which β^3^-homo-amino acids are used to strategically replace α-amino acids might be of interest as platforms for carbohydrate-based vaccines. That is because such mixed α/β-peptides adopt stable secondary structures closely related to those of natural α-peptides [21–22]. Moreover, inclusion of a single β-amino acid into an α-peptidic sequence already augments local and/or general stability against proteolytic degradation in vitro and in vivo; thus enabling the development of diverse peptidomimetics for an increasing number of applications [22–25]. Therefore we contemplated the use of mucin-derived α/β-hybrid glycopeptides as stable mimetics of naturally occurring glycocopeptide antigens for cancer vaccines. We were surprised to see how little precedence was available for this approach. Besides a recent report on α/δ-hybrid peptides derived from Neu2en and L-Glu [26], representing potentially immunogenic mimics of α-2,8-linked polysialic acid, only a few β-glycopeptides comprising *N*-acetylglucosamine [27–29] and *N*-acetylgalactosamine [30–31] (T_N_ antigen) linked to β^3^-homo-serine are known. Despite their importance as specific tumour antigens, conjugates of Fmoc-β^3^hSer and Fmoc-β^3^hThr carrying larger TACA structures such as the Thomsen–Friedenreich antigen (TF) or its sialylated variants (α2-6sTF and α2-3sTF) have not been reported to date.

**Figure 1:**
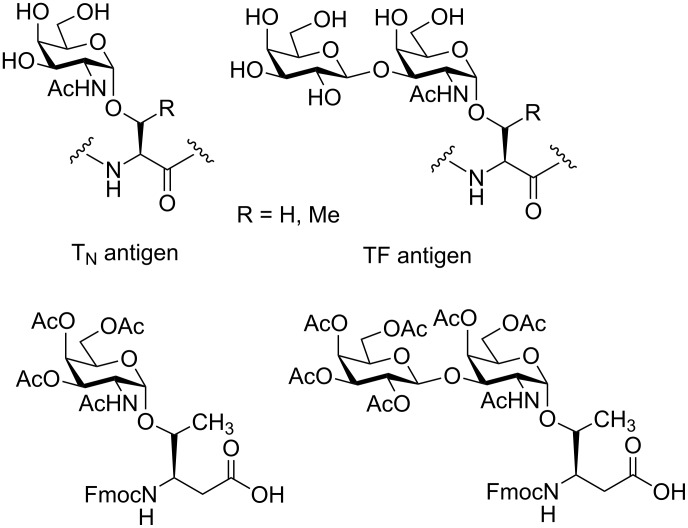
Structures of the naturally occurring T_N_ and TF antigens and the targeted Fmoc-β^3^hThr analogues.

By presenting orthogonally protected T_N_ and TF antigen conjugates of Fmoc-β^3^hThr (Figure 1) as well as a first α/β-hybrid glycopeptide analogue comprising the 20 amino acid tandem repeat sequence of the human mucin MUC1, we describe preliminary results of our synthetic efforts towards the preparation of mucin-type glycopeptide mimetics.

## Results and Discussion

**Scheme 1:**
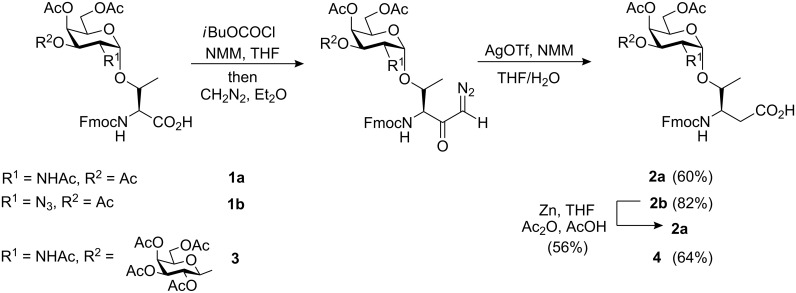
Synthesis of Fmoc-β^3^hThr antigen conjugates by Arndt–Eistert homologation.

Initial attempts to directly link the carbohydrate entity to the β-side chain of a preformed Fmoc-β^3^hThr conjugate were unsuccessful due to rapid lactonisation [29]. Similarly, despite the use of various glycosyl donors and reaction conditions, glycosylation of the corresponding dipeptide precursor Fmoc-β^3^hThr(OH)-Ala-OBn failed completely. Therefore we encountered the strategy of Arndt–Eistert homologation in the synthesis of the target Fmoc-β^3^hThr(αAc_3_GalNAc) and Fmoc-β^3^hThr(β(Ac_4_Gal(1–3))α(Ac_3_GalNAc)) conjugates **2a** and **4**, respectively, as reported by Norgren et al. [29]. T_N_ antigen derivative Fmoc-Thr(αAc_3_GalNAc)-OH (**1a**) was prepared according to published procedures [32–33] and converted into the corresponding diazo ketone upon treatment with isobutyl chloroformate in the presence of *N*-methylmorpholine (NMM) and diazomethane (Scheme 1). Without further purification, the latter was subjected to a silver-promoted Wolff-rearrangement, again using NMM as the base, providing the spectroscopically pure T_N_ antigen analogue Fmoc-β^3^hThr(αAc_3_GalNAc)-OH (**2a**) in 60% yield over two steps after aqueous work-up.

Compound **2a** was also accessible from a direct synthetic precursor of T_N_ derivative **1a** in which the 2-acetamido substituent was masked by an azido group. Thus, upon subjection of Fmoc-Thr(αAc_3_GalN_3_)-OH (**1b**) [32] to the same homologation sequence as before, the corresponding β^3^hThr analogue **2b** was obtained in 82% yield. Subsequent zinc-mediated reduction and acetylation led to the formation of conjugate **2a** in 56% yield.

During biosynthesis T_N_ antigen acts as an immediate precursor of the TF antigen. As a consequence, a biomimetic approach towards larger TACA structures via stepwise assembly of the glycan chain has been pursued in various antigen syntheses [33–34]. By applying chemical or enzymatic 3β-galactosylation, the 3-OH deprotected conjugate **2b** could be converted into the desired antigen derivative Fmoc-β^3^hThr(β(Ac_4_Gal(1–3))α(Ac_3_GalNAc))-OH (**4**). While this strategy certainly requires the use of optimised protecting group manipulations and glycosylation protocols, the alternative route to compound **4** via Arndt–Eistert homologation would benefit from an established and reliable synthesis of key building block **3** [32–33]. To our delight, the homologation reaction of glycosyl amino acid **3** again proceeded smoothly to afford the desired TF-β^3^hThr conjugate **4** in spectroscopically pure form and good chemical yield after aqueous work-up (Scheme 1).

**Scheme 2:**
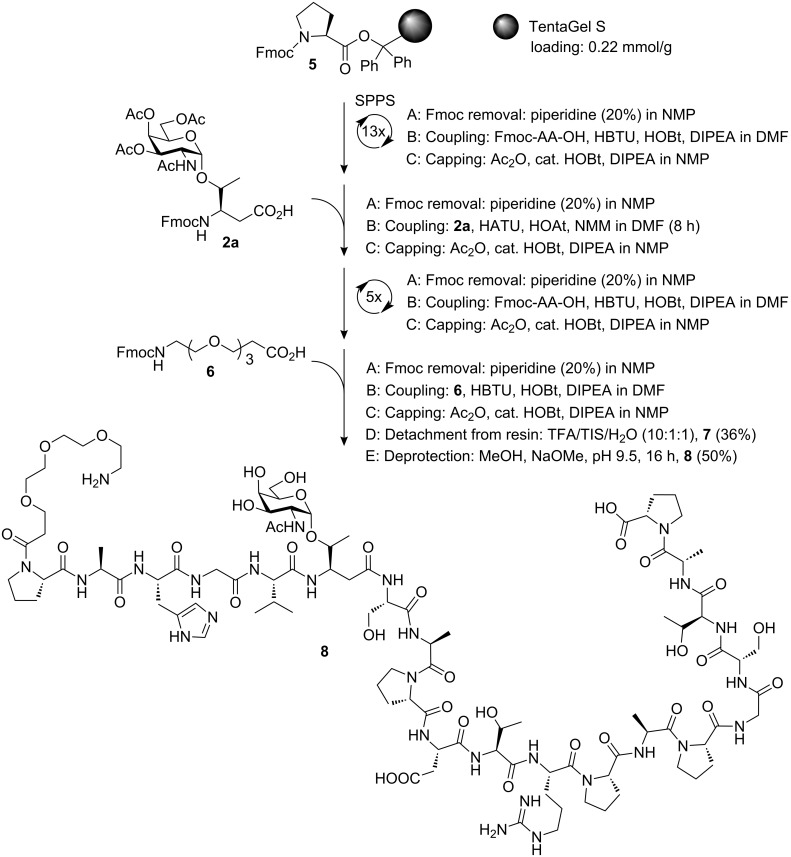
Solid-phase synthesis of the tumour-associated MUC1 α/β-hybrid glycopeptide analogue **8** carrying the T_N_ antigen glycan. NMP = *N*-methylpyrrolidone, HBTU = *O*-(1*H*-benzotriazol-1-yl)-*N*,*N*,*N*′,*N*′-tetramethyluronium hexafluorophosphate, HOBt = *N*-hydroxybenzotriazole, DIPEA = diisopropylethylamine, HATU = *O*-(7-azabenzotriazole-1-yl)-*N*,*N*,*N*′,*N*′-tetramethyluronium hexafluorophosphate, HOAt = *N*-hydroxy-7-azabenzotriazole, NMM = *N*-methylmorpholine, TIS = triisopropylsilane.

To demonstrate the usefulness of the novel glycosylated β^3^hThr conjugates as antigen mimics, T_N_ antigen analog **2a** was incorporated into an α/β-hybrid glycopeptide **7** comprising a full tandem repeat sequence of the epithelial mucin MUC1 and an *N*-terminal non-immunogenic triethylene glycol spacer. The latter can be used for further conjugation to immunostimulants (e.g., BSA [35] or tetanus toxoid [36]) and for immobilisation onto microarray platforms [37] within functional immunological studies. The MUC1 pseudo-glycopeptide was assembled in an automated synthesiser by the Fmoc-strategy on a TentaGel S resin **5** equipped with a bulky trityl linker [38] to avoid diketopiperazine formation and pre-loaded with Fmoc-proline (Scheme 2). The first 13 amino acids of the MUC1 sequence were coupled under standard conditions using piperidine in *N*-methylpyrrolidone (NMP) to remove the temporary Fmoc protecting group followed by coupling of excess (10 equiv) Fmoc-amino acid activated by HBTU/HOBt [39] and diisopropylethylamine (DIPEA) in DMF. Unreacted amino acids were capped after each cycle with Ac_2_O in the presence of DIPEA and catalytic amounts of HOBt in NMP. The sterically demanding glycosylated β^3^hthreonine building block **2a** (1.5 equiv), was coupled over an extended reaction time of 8 h employing the more reactive reagents HATU/HOAt [40] with *N*-methylmorpholine (NMM) in NMP for activation. After the final five Fmoc-amino acids of the TR-sequence were coupled according to the standard protocol, a triethylene glycol spacer **6** [41] (10 equiv) was attached using the standard coupling procedure, again. Simultaneous detachment of the glycopeptide from the resin and cleavage of the acid-labile amino acid side chain protective groups was achieved upon treatment with a mixture of TFA, triisopropylsilane and water. The resulting partially deblocked glycopeptide **7** was isolated after purification by semi-preparative RP-HPLC in a yield of 36%, based on the loaded resin **5**. The final de-*O*-acetylation of the glycan portion was accomplished upon prolonged treatment with catalytic amounts of NaOMe in methanol at pH 9.5 to afford glycopeptide **8** in 18% yield (based on the loaded resin) after semi-preparative RP-HPLC.

## Conclusion

Two novel tumour-associated carbohydrate antigen analogues with T_N_ and TF determinants *O*-glycosidically linked to the side chain of Fmoc-β^3^hThr-OH have been prepared by Arndt–Eistert homologation of the corresponding glycosylated α-amino acids **1a** and **1b**. The resulting β^3^hThr glycoconjugates are valuable antigen mimetics with potentially enhanced chemical and metabolic stability. They might serve as precursors for the preparation of further mucin-type antigen structures, e.g., sialylated ones or those based on core2 structures. In addition, the preparation of a first MUC1 pseudo-glycopeptide comprising the modified glycosyl amino acid Fmoc-β^3^hThr(αGalNAc)-OH at position Thr15 has been accomplished using solid-phase peptide synthesis. By appropriate conjugation, the resulting α/β-hybrid glycopeptide conjugate could be used as an antigen surrogate to elucidate the effects of chemically modified antibody determinants on the immunological properties of glycopeptide antigen analogues.

## Experimental

**General remarks:** DMF (amine-free, for peptide synthesis) and NMP were purchased from Roth and Ac_2_O in p.a. quality from Acros. Fmoc-protected amino acids were purchased from Orpegen Pharma. For solid-phase synthesis, pre-loaded TentaGel S resin (Rapp Polymere) was employed. Reactions were monitored by TLC with pre-coated silica gel 60 F_254_ aluminium plates (Merck KGaA, Darmstadt). HPLC analyses were performed on a JASCO-HPLC system with Phenomenex Luna C18(2) (250 × 4.6 mm, 10 μm) and Phenomenex Jupiter C18(2) (250 × 4.6 mm, 10 μm) columns at a flow rate of 1 mL min^−1^. Preparative RP-HPLC separation was carried out on a JASCO-HPLC System with Phenomenex Luna C18(2) (250 × 30 mm, 10 μm) and Phenomenex Jupiter C18(2) (250 × 30 mm, 10 μm) columns at a flow rate of 20 mL min^−1^ or 10 mL min^−1^. ^1^H, ^13^C and 2D NMR spectra were recorded on a Bruker AC-300 or a Bruker AM-400 spectrometer. The chemical shifts are reported in ppm relative to the signal of the deuterated solvent. Multiplicities are given as: s (singlet), br s (broad singlet), d (doublet), t (triplet) and m (multiplet). In the case of known compounds, all spectra obtained were consistent with the literature. HR-ESI-mass spectra were recorded on a Micromass Q TOF Ultima 3 spectrometer and optical rotations were measured at 546 nm with a Perkin-Elmer polarimeter 241.

### General procedure for the synthesis of diazomethane in ethereal solution

**Caution:** Diazomethane is toxic, highly-volatile, cancerogenic and explosive. Its generation and handling thus requires special precautions [42]. With regard to the free acids, 10 equiv of *N*-methyl-*N*-nitroso-urea were added to a solution of 50% KOH in H_2_O which was layered on top with Et_2_O. The organic layer was decanted and replaced by a new portion of Et_2_O until the organic layer was no longer coloured. The united organic phases were dried over KOH at −25 °C for 3 h.

**General procedure (GP1) for the synthesis of diazo ketones:** The free acid (1 equiv) was dissolved in 2 mL of dry THF under an argon atmosphere. At −25 °C, 1 equiv of NMM and 1 equiv of isobutyl chloro formate were added subsequently and the resulting suspension was stirred for 20 min at this temperature. The mixture was allowed to reach 0 °C and the diazomethane solution in Et_2_O was added. The yellow solution was stirred 20 min at 0 °C before it was allowed to reach room temperature and stirred for further 16 h. Excess of diazomethane was destroyed by adding a few drops of acetic acid to the solution until no further nitrogen formation was observed. The solvents were removed under reduced pressure and the residue was dissolved in 20 mL Et_2_O. The organic layer was washed twice with saturated aqueous NaHCO_3_, saturated aqueous NH_4_Cl and brine. The organic layer was dried over Na_2_SO_4_, filtered, and the solvents were removed under reduced pressure. The resulting diazo ketones were used without further purification.

**General procedure (GP2) for the Wolff-rearrangement:** The diazo ketone (1 equiv) was dissolved in a mixture of THF/H_2_O (9:1) and was cooled to 0 °C. A solution of 0.11 equiv silver trifluoroacetate in 2.3 equiv NMM was added, and the mixture was stirred for 16 h while it was allowed to warm to room temperature. After evaporation of THF, the aqueous layer was diluted with saturated aqueous NaHCO_3_ and Et_2_O was added. The organic layer was extracted three times with saturated aqueous NaHCO_3_. The aqueous phases were collected, cooled to 0 °C and acidified to pH 1. The resulting suspension was extracted five times with Et_2_O. The organic layer was dried over Na_2_SO_4_ and evaporated in vacuo.

***N*-(9*H*-fluoren-9-yl)methoxycarbonyl-[α-3,4,6-tri-*O*-acetyl-2-acetamido-2-deoxy-galactopyranosyl]-β^3^-homo-threonine 2a:** ***Procedure A:*** The synthesis followed the general procedures GP1 and GP2. Amounts: 150 mg (0.22 mmol) **1a**. Yield: 90 mg (0.13 mmol), 60%, colourless amorphous solid. Analytical RP-HPLC (Luna, MeCN–H_2_O + 0.1% TFA, 20:80 → 60:40, 40 min, *t*_R_ = 29.8 min). ***Procedure B:*** To a stirred solution of **2b** (860 mg, 0.48 mmol) in a mixture of THF–Ac_2_O–AcOH (3:2:1, 36 mL) activated zinc dust (0.6 g, 9.1 mmol) was added. Activation was achieved by suspension in aq. 2% solution of CuSO_4_, followed by subsequent washings with water, EtOAc and Et_2_O. The reaction mixture was stirred for 16 h at room temperature, diluted with 50 mL THF and filtered through Hyflo Supercel^®^. The filtrate was concentrated in vacuo and co-evaporated five times with toluene and CH_2_Cl_2_. The residue was dissolved in 50 mL CH_2_Cl_2_, washed with saturated aqueous NaHCO_3_ and brine, dried (Na_2_SO_4_) and concentrated in vacuo. Yield: 328 mg (0.48 mmol), 56%, colourless amorphous solid; [α]_D_^23^ = 42.6 (*c* = 1.00, CHCl_3_); ^1^H NMR (COSY, HSQC, 400 MHz, CD_3_OD), δ (ppm) = 7.81 (d, 2 H, *J* = 7.2 Hz, Fmoc-H4, Fmoc-H5), 7.66, 7.65 (2d, 2 H, *J* = 7.6 Hz, Fmoc-H1, Fmoc-H8), 7.39 (pt, 2 H, Fmoc-H3, Fmoc-H6), 7.32 (t, 2 H, *J* = 7.6 Hz, Fmoc-H2, Fmoc-H7), 5.44 (d, 1 H, *J* = 2.4 Hz, GalNAc-H4), 5.10 (dd, 1 H, *J* = 3.2 Hz, 11.6 Hz, GalNAc-H3), 4.98 (d, 1 H, *J* = 3.6 Hz, GalNAc-H1), 4.56 (pdd, 1 H, Fmoc-CH_2_), 4.45 (dd, 1 H, *J* = 3.6 Hz, 11.6 Hz, GalNAc-H2), 4.41–4.35 (m, 1 H, Fmoc-CH_2_), 4.31 (pt, 1 H, GalNAc-H5), 4.21 (pt, 1 H, Fmoc-H9), 4.15–4.07 (m, 2 H, GalNAc-H6), 4.05–4.02 (m, 1 H, hT^β^), 3.83 (pdd, 1 H, hT^γ^), 2.55 (pdd, 1 H, hT^α^), 2.38 (pdd, 1 H, hT^α^), 1.76 (d, 3 H, *J* = 6.4 Hz, hT^δ^); ^13^C NMR (DEPT, HSQC, 100.6 MHz, CD_3_OD), δ (ppm) = 175.1, 172.2, 170.7, 170.7, 170.6 (C=O), 157.0 (C=O-urethane), 144.0, 143.8 (Fmoc-C1a, Fmoc-C8a), 141.3, 141.3 (Fmoc-C4a, Fmoc-C5a), 127.4 (Fmoc-C3, Fmoc-C6), 126.8, 126.7 (Fmoc-C2, Fmoc-C7), 124.7, 124.6 (Fmoc-C1, Fmoc-C8), 119.6, 119.5 (Fmoc-C4, Fmoc-C5), 99.1 (GalNAc-C1), 77.6 (hT^γ^), 68.3 (GalNAc-C3), 67.4 (GalNAc-C4), 66.6 (GalNAc-C5), 65.9 (Fmoc-CH_2_), 61.8 (GalNAc-C6), 52.6 (hT^β^), 47.6 (GalNAc-C2), 47.3 (Fmoc-C9), 36.8 (hT^α^), 21.3 (CH_3_-NHAc), 19.3, 19.2, 19.1 (CH_3_-Ac), 16.7 (hT^δ^); HR-ESI-MS (positive, *m/z*): 707.2433 ([M+Na]^+^, calc.: 707.2428).

***N*-(9*H*-fluoren-9-yl)methoxycarbonyl-[α-3,4,6-tri-*O*-acetyl-2-azido-2-deoxy-galactopyranosyl]-β^3^-homo-threonine 2b:** The synthesis followed the general procedures GP1 and GP2. Amounts: 156 mg (0.23 mmol) **1b**. Yield: 130 mg (0.19 mmol), 82%, colourless amorphous solid; [α]_D_^23^ = 56.0 (*c* = 1.00, CHCl_3_); ^1^H NMR (400 MHz, COSY, HSQC, CD_3_OD), δ (ppm) = 7.79 (d, 2 H, *J* = 7.6 Hz, Fmoc-H4, Fmoc-H5), 7.66 (d, 2 H, *J* = 7.6 Hz, Fmoc-H1, Fmoc-H8), 7.39 (t, 2 H, *J* = 7.2 Hz, Fmoc-H3, Fmoc-H6), 7.32 (t, 2 H, *J* = 7.2 Hz, Fmoc-H2, Fmoc-H7), 5.44 (d, 1 H, *J* = 2.0 Hz, GalNAc-H4), 5.33 (dd, 1 H, *J* = 3.2 Hz, 11.2 Hz, GalNAc-H3), 5.14 (d, 1 H, *J* = 3.6 Hz, GalNAc-H1), 4.41–4.32 (m, 3 H, Fmoc-CH_2_, Fmoc-H9), 4.21 (pt, 1 H, GalNAc-H5), 4.14–4.06 (m, 3 H, GalNAc-H6, hT^β^), 3.91–3.86 (m, 2 H, GalNAc-H2, hT^γ^), 2.76 (dd, 1 H, *J* = 5.2 Hz, 16 Hz, hT^α^), 2.55 (dd, 1 H, *J* = 8.8 Hz, 16.4 Hz, hT^α^), 2.14, 2.02, 1.98 (3s, 9 H, 3 × CH_3_-Ac); 1.76 (d, 3 H, *J* = 6.4 Hz, hT^δ^); ^13^C NMR (101 MHz, DEPT, HSQC, CD_3_OD), δ (ppm) = 173.5, 170.7, 170.6, 170.1 (C=O), 157.1 (C=O-urethane), 144.0, 143.9 (Fmoc-C1a, Fmoc-C8a), 141.2 (Fmoc-C4a, Fmoc-C5a), 127.4 (Fmoc-C3, Fmoc-C6), 126.8, 126.7 (Fmoc-C2, Fmoc-C7), 124.9 (Fmoc-C1, Fmoc-C8), 119.5 (Fmoc-C4, Fmoc-C5), 98.6 (GalNAc-C1), 77.5 (hT^γ^), 69.0 (GalNAc-C3), 67.7 (GalNAc-C4), 66.8 (GalNAc-C5), 66.4 (Fmoc-CH_2_), 58.4 (GalNAc-C6), 52.0 (hT^γ^), 47.6 (GalNAc-C2), 47.3 (Fmoc-C9), 34.8 (hT^α^), 19.3, 19.2, 19.1 (CH_3_-Ac), 16.4 (hT^δ^); HR-ESI-MS (positive, *m/z*): 691.2232 ([M+Na]^+^ calc.: 691.2227).

***N*-(9*H*-fluoren-9-yl)methoxycarbonyl-[(β-2,3,4,6-tetra-*O*-acetylgalactopyranosyl)-(1–3)-(α-2-acetamido-4,6-di-*O*-acetyl-2-deoxy-galactopyranosyl)]-β^3^-homo-threonine 4:** The synthesis followed the general procedures GP1 and GP2. Amounts: 200 mg (0.21 mmol) **3**. Yield: 130.3 mg (0.13 mmol), 64%, colourless amorphous solid; [α]_D_^23^ = 29.9 (*c* = 0.50, CHCl_3_); ^1^H NMR (400 MHz, COSY, HSQC, CD_3_OD), δ (ppm) = 7.85 (d, 2 H, *J* = 7.6 Hz, Fmoc-H4, Fmoc-H5), 7.69 (d, 2 H, *J* = 7.6 Hz, Fmoc-H1, Fmoc-H8), 7.44 (t, 2 H, *J* = 7.6 Hz, Fmoc-H3, Fmoc-H6), 7.36 (t, 2 H, *J* = 7.6 Hz, Fmoc-H2, Fmoc-H7), 5.43 (d, 1 H, *J* = 3.4 Hz, GalNAc-H4), 5.39 (m, 1 H, Gal-H3), 5.04–5.02 (m, 2 H, Gal-H4, Gal-H2), 4.98 (under H_2_O-peak, GalNAc-H1), 4.71 (m, 1H, Gal-H1), 4.51 (d, 2 H, *J* = 6.2 Hz, Gal-H6), 4.44 (dd, 1 H, *J* = 3.5 Hz, 11.5 Hz, GalNAc-H2), 4.28–4.23 (m, 2 H, Gal-H5, Fmoc-H9), 4.23–4.21 (m, 3 H, Fmoc-CH_2_, GalNAc-H6), 4.09–3.91 (m, 4 H, GalNAc-H5, Fmoc-CH_2_, hT^β^, GalNAc-H3), 3.86 (dd, 1 H, *J* = 2.7 Hz, 6.2 Hz, hT^γ^), 2.71 (dd, 1 H, *J* = 6.3 Hz, 15.5 Hz, hT^α^), 2.55 (dd, 1 H, *J* = 8.7 Hz, 15.6 Hz, hT^α^), 2.17, 2.15, 2.06, 2.06, 2.05, 2.04, 1.97 (7s, 21 H, 6 × CH_3_-Ac, CH_3_-AcNH), 1.22 (d, 3 H, *J* = 6.6 Hz, hT^δ^); ^13^C NMR (101 MHz, DEPT, HSQC, CD_3_OD), δ (ppm) = 174.7, 173.0, 172.5, 172.2, 171.6, 171.2 (C=O), 158.5 (C=O-urethane), 145.5, 145.3 (Fmoc-C1a, Fmoc-C8a), 142.8, 142.8 (Fmoc-C4a, Fmoc-C5a), 129.0, 128.4 (Fmoc-C3, Fmoc-C6), 126.3, 126.2 (Fmoc-C2, Fmoc-C7), 121.2 (Fmoc-C1, Fmoc-C8, Fmoc-C4, Fmoc-C5), 102.6 (Gal-C1), 100.6 (GalNAc-C1), 78.2 (hT^γ^), 74.9 (GalNAc-C3), 72.2 (Gal-C4), 71.8 (GalNAc-C5), 71.2 (GalNAc-C4), 70.1 (Gal-C2), 68.8 (Gal-C5), 68.5 (Gal-C3), 67.4 (Gal-C6), 64.3 (Fmoc-CH_2_), 62.3 (GalNAc-C6), 53.6 (hT^β^), 50.3 (GalNAc-C2), 36.5 (hT^α^), 23.0 (CH_3_-NHAc), 20.8, 20.8, 20.7, 20.5 (CH_3_-Ac), 18.0 (hT^δ^); HR-ESI-MS (positive, *m/z*): 1159.3216 ([M+Na]^+^, calc.: 1159.3210).

**Amino-4,7,10-trioxadodecanylamido-*N*-L-prolyl-L-alanyl-L-histidyl-L-glycyl-L-valyl-*O*-[α-3,4,6-tri-*O*-acetyl-2-acetamido-2-deoxy-galactopyranosyl]-homo-β^3^-threonyl-L-seryl-L-alanyl-L-prolyl-L-aspartyl-L-threonyl-L-arginyl-L-prolyl-L-alanyl-L-prolyl-L-glycyl-L-seryl-L-threonyl-L-alanyl-L-proline 7:** The synthesis was carried out in an Applied Biosystems ABI 433A peptide synthesiser (standard program Fastmoc 0.1 mmol) using pre-loaded Fmoc-Pro-Trt-TentaGel S resin (455 mg, 0.10 mmol; loading: 0.22 mmol/g). For the coupling reactions, the amino acids Fmoc-Ala-OH, Fmoc-Arg(Pmc)-OH, Fmoc-Asp-OH, Fmoc-Gly-OH, Fmoc-His(Trt)-OH, Fmoc-Pro-OH, Fmoc-Ser(*t*Bu)-OH, Fmoc-Thr(*t*Bu)-OH, and Fmoc-Val-OH were employed. In every coupling cycle, the *N*-terminal Fmoc group was removed by treatment of the resin with a solution of piperidine (20%) in NMP for at least 3 × 2.5 min. The coupling of the amino acids (1 mmol or 10 equiv based on the loaded resin) was carried out with HBTU (1 mmol), HOBt (1 mmol) and DIPEA (2 mmol) in DMF (20–30 min vortex). After every coupling step, unreacted amino groups were capped by treatment with a mixture of Ac_2_O (0.5 M), DIPEA (0.125 M) and HOBt (0.015 M) in NMP (10 min vortex). Coupling of the glycosylated β^3^hThr building block **2a** (102.70 mg, 0.15 mmol) was performed using HATU (1.2 equiv with respect to the glycosyl amino acid), HOAt (1.2 equiv) and NMM (4 equiv) for activation (8 h vortex). After coupling of the remaining five amino acids by the standard procedure, the triethylene glycol spacer (1 mmol, 10 equiv based on the loaded resin) was coupled using HBTU (1 mmol), HOBt (1 mmol) and DIPEA (2 mmol) in DMF (20–30 min vortex) and the *N*-terminal Fmoc group was removed by piperidine (20%) in NMP. Detachment from the resin and simultaneous removal of all side chain protecting groups was performed in a Merrifield glass reactor by shaking with TFA (10 mL), TIS (1.0 mL) and H_2_O (1.0 mL) for 3 h. The solution was filtered, the resin was washed with CH_2_Cl_2_ (5 × 10 mL) and the combined solutions were concentrated in vacuo to a volume of 0.5 mL. After co-evaporation with toluene (3 × 10 mL), the crude product was dissolved in H_2_O and subjected to lyophilisation. The crude product was purified by RP-HPLC (Luna, MeCN–H_2_O + 0.1% TFA, 5:95 → 100:0, 50 min, *t*_R_= 25.2 min). Yield: 88.3 mg (0.04 mmol), 36%, colourless amorphous solid; [α]_D_^23^ = −79.9 (*c* = 1.00, H_2_O); ^1^H NMR (400 MHz, COSY, HSQC, TOCSY, D_2_O), δ (ppm): 8.50 (m, 1 H, H^ε^), 7.19 (m, 1 H, H^δ^), 5.31–5.28 (m, 1 H, GalNAc-H4), 5.08 (dd, 1 H, *J* = 3.1 Hz, 11.7 Hz, GalNAc-H3), 5.01 (d, 1 H, *J* = 3.5 Hz, GalNAc-H1), 4.64–4.54 (m, 2 H, D^α^ {4.61}, H^α^ {4.57}), 4.54–4.37 (m, 5 H, R^α^ {4.52}, A_2_^α^ {4.47}, A_3_^α^ {4.46}, A_4_^α^ {4.45}, S_1_^α^ {4.39, t, *J* = 5.5 Hz}), 4.36–4.20 (m, 10 H, S_2_^α^ {4.33}, hT^β^ {4.27}, A_1_^α^ {4.29}, GalNAc-H5 {4.32}, T_1_^α^ {4.30}, T_2_^α^ {4.24}, P_1–4_^α^ {4.29} {4.28} {4.27} {4.24}), 4.19 (d, 1 H, *J* = 4.5 Hz, V^α^), 4.15–3.89 (m, 6 H, GalNAc-H2 {4.12}, T_1_^β^, T_2_^β^ {4.09} {4.07}, GalNAc-H6 {4.02}, P_5_^α^ {3.94}), 3.89–3.73 (m, 7 H, G_1_^α^ {3.86}, G_2_^α^ {3.83}, G_1_^α^ {3.80}, G_2_^α^ {3.77}, hT^γ^ {3.77}, S_2_^β^ {3.76}), 3.73–3.59 (m, 11 H, P_1–5_^δ^ {3.67}, S_1_^β^ {3.65}, 2 × CH_2_-spacer {3.64} {3.62}), 3.59–3.28 (m, 13 H, 2 × CH_2_-spacer {3.57}, 2 × CH_2_-spacer {3.54}, P_1–5_^δ^ {3.52}), 3.18 (dd, 1 H, *J* = 5.2 Hz, 15.1 Hz, H^β^), 3.11–3.01 (m, 5 H, CH_2_-spacer {3.08}, H^β^ {3.07}, R^δ^ {3.07}), 2.84 (dd, 1 H, *J* = 6.5 Hz, 16.8 Hz, D^β^), 2.75 (dd, 1 H, *J* = 6.5 Hz, 17.3 Hz, D^β^), 2.67–2.40 (m, 4 H, CH_2_-spacer {2.61} {2.55}, hT^α^ {2.59} {2.46}), 2.23–2.05 (m, 8 H, P_1–5_^β^ {2.20} {2.17} {2.14}, CH_3_-Ac {2.07}), 2.01–1.66 (m, 29 H, 4 × CH_3_-Ac {1.94} {1.92} {1.89} {1.88}, P_1–5_^γ^ {1.95–1.83}), P_1–5_^β^ {1.92–1.71}, V^β^ {1.86}, R^β^ {1.70}), 1.65–1.46 (m, 3 H, R^β^ {1.61}, R^γ^ {1.54}), 1.28–1.17 (m, 12 H, A_1–4_^β^ {1.24} {1.20}), 1.07–1.01 (m, 9 H, T_1_^γ^ {1.07}, hT^δ^ {1.06}, T_2_^γ^ {1.04}), 0.76 (d, 3 H, *J* = 5.8 Hz, V^γ^), 0.71 (d, 3 H, *J* = 6.4 Hz, V^γ^); ^13^C NMR (101 MHz, DEPT, HSQC, D_2_O), δ (ppm) = 176.6, 175.7, 174.9, 174.4, 174.3, 173.9, 173.43, 173.4, 173.1, 172.9, 172.8, 172.6, 172.4, 172.3, 171.9, 171.5, 171.3, 171.2, 171.1, 170.9, 170.8, 170.7 (C=O), 163.3, 163.0, 162.6, 162.2 (TFA), 156.7 (C=NH), 133.5 (H^β^), 128.4 (H^ε^), 117.3 (H^δ^), 98.2, 98.0 (GalNAc-C1), 77.2, 76.9 (hT^γ^), 69.6, 69.5, 69.4, 69.4 (CH_2_-Spacer), 68.4 (GalNAc-C3), 68.0 (GalNAc-C2), 67.0 (T_1_^β^, T_2_^β^), 66.3, 66.0 (CH_2_-spacer), 62.5 (GalNAc-C6), 61.5, 61.1 (S_1_^β^, S_2_^β^), 60.8, 60.5, 60.0, 59.7, 58.7 (P_1–5_^α^), 59.6, 59.3 (T_1_^α^, T_2_^α^), 58.9 (V^α^), 55.5, 54.9, 52.24 (H^α^), 51.1 (R^α^), 50.5 (hT^β^), 50.0 (D^α^), 49.6 (GalNAc-C2), 48.0, 47.9, 47.7, 47.4 (P_1–5_^δ^), 47.8, 47.8, 47.6, 47.6 (A_1_^α^, A_2_^α^, A_3_^α^, A_4_^α^), 42.4, 42.3 (G_1_^α^, G_2_^α^), 40.5 (CH_2_-spacer), 39.0 (R^δ^), 36.2 (hT^α^), 34.9 (D^β^), 34.0 (CH_2_-spacer), 29.9, 29.8 (V^β^), 29.6, 29.3, 29.2, 29.2, 28.1 (P_1–5_^β^), 27.4 (R^β^), 26.2 (H^β^), 24.7, 24.6, 24.5, 24.3 (P_1–5_^γ^), 23.9 (R^γ^), 21.8 (CH_3_-AcNH), 20.3, 20.0, 20.0 (CH_3_-Ac) , 18.8, 18.7 (T_1_^γ^, T_2_^γ^), 18.3, 17.7 (V^γα^ V^γβ^), 16.6 (hT^δ^), 16.2, 15.4, 15.2, 15.1 (A_1–4_^β^); HR-ESI-MS: 1217.0941 ([M+2H]^+^, calc.: 1217.0932).

**Amino-4,7,10-trioxadodecanylamido-*N*-L-prolyl-L-alanyl-L-histidyl-L-glycyl-L-valyl-*O*-[α-2-acetamido-2-deoxy-galactopyranosyl]-homo-β^3^-threonyl-L-seryl-L-alanyl-L-prolyl-L-aspartyl-L-threonyl-L-arginyl-L-prolyl-L-alanyl-L-prolyl-L-glycyl-L-seryl-L-threonyl-L-alanyl-L-proline 8:** Peptide **7** was dissolved in 10 mL of methanol (HPLC grade). A fresh solution of sodium methanolate in methanol (0.5 g Na in 25 mL methanol (HPLC grade)) was added drop wise until pH 9.5 was reached. The reaction mixture was stirred over night and neutralised with a few drops of acetic acid. The solvent was removed in vacuo and the residue was dissolved in H_2_O and subjected to lyophilisation. The crude product was purified by RP-HPLC (Luna, MeCN–H_2_O + 0.1% TFA, 5:95 → 100:0, 60 min, *t*_R_ = 15.3 min). Yield: 44 mg (0.02 mmol), 50% (18% based on the loaded resin **5**), colourless amorphous solid; [α]_D_^23^ = −83.7 (*c* = 1.00, H_2_O); ^1^H NMR (400 MHz, COSY, HSQC, D_2_O), δ (ppm) = 8.50 (d, 1 H, *J* = 1.6 Hz, H^δ^), 7.20 (d, 1 H, *J* = 1.3 Hz, H^ε^), 4.89 (d, 1 H, *J* = 3.9 Hz, GalNAc-H1), 4.62 (t, 1 H, *J* = 6.8 Hz, D^α^), 4.58 (dd, 1 H, *J* = 6.7 Hz, 8.6 Hz, H^α^), 4.55–4.49 (m, 1 H, R^α^), 4.50–4.43 (m, 3 H, A_1–3_^α^ {4.48} {4.47} {4.45}), 4.40 (t, 1 H, *J* = 5.4 Hz, S_1_^α^), 4.34–4.21 (m, 7 H, S_2_^α^ {4.32}, P_1–5_^α^ {4.29} {4.28} {4.28} {4.26} {4.26}, T_1_^α^{4.24}), 4.20 (d, 1 H, *J* = 4.5 Hz, V^α^), 4.16–4.10 (m, 1 H, A_4_^α^), 4.11–4.05 (m, 2 H, T_1_^β^, T_2_^β^), 4.00 (dd, 1 H, *J* = 3.8 Hz, 11.1 Hz, GalNAc-H2), 3.95–3.89 (m, 2 H, T_2_^α^ {3.93}, GalNAc-H5 {3.89}), 3.89–3.82 (m, 4 H, G_1_^α^ {3.87}, G_1_^α^ {3.84}, G_2_^α^ {3.84}, GalNAc-H4 {3.84}), 3.82–3.74 (m, 3 H, GalNAc-H3 {3.80}, G_2_^α^ {3.80}, S_1_^β^ {3.79}), 3.77–3.74 (m, 1 H, S_1_^β^), 3.74–3.61 (m, 11 H, P_1–5_^δ^ {3.70} {3.68} {3.68} {3.68} {3.67}, GalNAc-H6 {3.65}, 2 × CH_2_-spacer {3.65} {3.62}), 3.61–3.31 (m, 15 H, S_2_^β^ {3.59}, 4 × CH_2_-spacer {3.58}, 4 × CH_2_-spacer {3.54}, P_1–5_^δ^ {3.58–3.48}), 3.19 (dd, 1 H, *J* = Hz, H^α^), 3.11–3.02 (m, 5 H, CH_2_-spacer {3.09}, R^δ^ {3.07}, H^α^ {3.07}), 2.85 (dd, 1 H, *J* = 6.6 Hz, 17.4 Hz, D^β^), 2.76 (dd, 1 H, *J* = 6.6 Hz, 17.0 Hz, D^β^), 2.69–2.38 (m, 4 H, CH_2_-spacer {2.62}, hT^α^ {2.57}, CH_2_-spacer{2.55}, hT^α^ {2.43}), 2.26–2.07 (m, 5 H, P_1–5_^β^ {2.20} {2.18} {2.17} {2.16} {2.14}), 2.00–1.48 (m, 23 H, P_1–5_^γ^ {1.98–1.80}, P_1–5_^β^ {1.92–1.70}, CH_3_-AcNH {1.93}, V^β^ {1.85}, R^β^ {1.71}, R^β^ {1.61}, R^γ^ {1.54}), 1.29–1.18 (m, 12 H, A_1–4_^β^ {1.26}, {1.24}, {1.21}, {1.21}), 1.10–1.02 (m, 9 H, T_1_^γ^, T_2_^γ^, hT^δ^ {1.06}, {1.04}, {1.04}), 0.76 (d, 3 H, *J* = 6.7 Hz, V^γ^), 0.72 (d, 3 H, *J* = 6.7 Hz, V^γ^); ^13^C NMR (101 MHz, DEPT, HSQC, D_2_O), δ (ppm) = 175.9, 174.9, 174.4, 174.4, 173.9, 173.5, 173.5, 173.1, 172.9, 172.8, 172.6, 172.5, 172.4, 172.4, 172.0, 171.5, 171.3, 171.2, 171.1, 170.9, 170.8 (C=O), 163.1, 162.7 (TFA), 156.7 (C=NH), 133.4 (H^β^), 128.4 (H^ε^), 117.3 (H^δ^), 98.0 (GalNAc-C1), 76.3 (hT^γ^), 69.6, 69.5, 69.5, 69.4 (CH_2_-Spacer), 71.3 (GalNAc-C5), 67.4 (GalNAc-C3), 61.5 (GalNAc-C6), 61.2, 61.1 (S_1_^β^, S_2_^β^), 60.8, 60.5, 60.0, 59.7, 58.7 (P_1–5_^α^), 59.6, 59.3 (T_1_^α^, T_2_^α^), 58.9 (V^α^), 55.5 (S_2_^α^), 54.9 (S_1_^α^), 52.3 (H^α^), 51.1 (R^α^), 50.5 (hT^β^), 50.1 (D^α^), 50.0 (GalNAc-C2), 49.6 (A_4_^α^), 48.0, 47.9, 47.7, 47.4 (P_1–5_^δ^), 47.8, 47.6, 47.6 (A_1_^α^, A_2_^α^, A_3_^α^), 42.4, 42.3 (G_1_^α^, G_2_^α^), 40.5 (CH_2_-spacer), 39.0 (R^δ^), 36.1 (hT^α^), 35.0 (D^β^), 34.0 (CH_2_-spacer), 29.9 (V^β^), 29.6, 29.3, 29.2, 29.1, 28.7 (P_1–5_^β^), 27.4 (R^β^), 26.2 (H^β^), 24.7, 24.6, 24.5, 24.3 (P_1–5_^γ^), 23.9 (R^γ^), 21.9 (CH_3_-AcNH), 18.8, 18.7 (T_1_^γ^, T_2_^γ^), 18.3, 17.7 (V^γα^, V^γβ^), 16.6 (hT^δ^), 16.2, 15.4, 15.1, 15.0 (A_1–4_^β^); HR-ESI-MS: 1154.0817 ([M+2H]^2+^, calc.: 1154.0774).

## Supporting Information

File 1 NMR spectra of compounds **2a**, **2b**, **4**, **7**, **8** and HPLC chromatogram of compound **2a**.

## References

[1] Dwek R A (1996). Chem Rev.

[2] Lis H, Sharon N (1993). Eur J Biochem.

[3] Varki A (1993). Glycobiology.

[4] Pratt M R, Bertozzi C R (2005). Chem Soc Rev.

[5] Taylor-Papadimitriou J, Epenetos A A (1994). Trends Biotechnol.

[6] Danishefsky S J, Allen J R (2000). Angew Chem, Int Ed.

[7] Slovin S S, Keding S J, Ragupathi G (2005). Immunol Cell Biol.

[8] Becker T, Dziadek S, Wittrock S, Kunz H (2007). Curr Opin Mol Ther.

[9] Liakatos A, Kunz H (2007). Curr Opin Mol Ther.

[10] Buskas T, Thompson P, Boons G-J (2009). Chem Commun.

[11] Urban D, Skrydstrup T, Beau J-M (1998). Chem Commun.

[12] Röhrig C H, Takhi M, Schmidt R R (2001). Synlett.

[13] Kuberan B, Sikkander S A, Tomiyama H, Linhardt R J (2003). Angew Chem, Int Ed.

[14] Cipolla L, Rescigno M, Leone A, Peri F, La Ferla B, Nicotra F (2002). Bioorg Med Chem.

[15] Rich J J, Bundle D R (2004). Org Lett.

[16] Bousquet E, Spadaro A, Pappalardo M S, Bernardini R, Romeo R, Panza L, Ronsisvalle G (2000). J Carbohydr Chem.

[17] Bundle D R, Rich J J, Jacques S, Yu H N, Nitz M, Ling C-C (2005). Angew Chem, Int Ed.

[18] Rich J J, Wakarchuk W W, Bundle D R (2006). Chem–Eur J.

[19] Wu X, Lipinski T, Paszkiewicz E, Bundle D R (2008). Chem–Eur J.

[20] Mersch C, Wagner S, Hoffmann-Röder A (2009). Synlett.

[21] Hook D F, Bindschädler P, Mahajan Y R, Šebesta R, Kast P, Seebach D (2005). Chem Biodiversity.

[22] Aguilar M-I, Purcell A W, Devi R, Lew R, Rossjohn J, Smith A I, Perlmutter P (2007). Org Biomol Chem.

[23] Cheng R P, Gellman S H, DeGrado W F (2001). Chem Rev.

[24] Steer D L, Lew R A, Perlmutter P, Smith A I, Aguilar M-I (2002). Curr Med Chem.

[25] Seebach D, Gardiner J (2008). Acc Chem Res.

[26] Saludes J P, Ames J B, Gervay-Hague J (2009). J Am Chem Soc.

[27] Disney M D, Hook D F, Namoto K, Seeberger P H, Seebach D (2005). Chem Biodiversity.

[28] Norgren A S, Geitmann M, Danielson U H, Arvidsson P I (2007). J Mol Recognit.

[29] Norgren A S, Norberg T, Arvidsson P I (2007). J Pept Sci.

[30] Norgren A S, Arvidsson P I (2005). Org Biomol Chem.

[31] Norgren A S, Arvidsson P I (2008). J Org Chem.

[32] Paulsen H, Hölck J-P (1982). Carbohydr Res.

[33] Brocke C, Kunz H (2004). Synthesis.

[34] Dziadek S, Brocke C, Kunz H (2004). Chem–Eur J.

[35] Dziadek S, Kowalczyk D, Kunz H (2005). Angew Chem, Int Ed.

[36] Kaiser A, Gaidzik N, Westerlind U, Kowalczyk D, Hobel A, Schmitt E, Kunz H (2009). Angew Chem, Int Ed.

[37] Westerlind U, Schröder H, Hobel A, Gaidzik N, Kaiser A, Niemeyer C M, Schmitt E, Waldmann H, Kunz H (2009). Angew Chem, Int Ed.

[38] Fréchet J M J, Haque K E (1975). Tetrahedron Lett.

[39] Dourtoglou V, Gross B, Lambropoulou V, Zioudrou C (1984). Synthesis.

[40] Carpino L A (1993). J Am Chem Soc.

[41] Keil S, Claus C, Dippold W, Kunz H (2001). Angew Chem, Int Ed.

[42] Lombardi P (1994). Chem Ind (London).

