# Supplementary material for: Synthesis of glycosylated β3-homo-threonine conjugates for mucin-like glycopeptide antigen analogues
Source: Beilstein J Org Chem. 2010 May 12;6:47. doi: 10.3762/bjoc.6.47 (PMC2887299; doi:10.3762/bjoc.6.47)
Supplement: File 1 — NMR spectra of compounds 2a, 2b, 4, 7, 8 and HPLC chromatogram of compound 2a. [file Beilstein_J_Org_Chem-06-47-s001.pdf]

Supporting Information

for

Synthesis of glycosylated β^3-homo-threonine conjugates for mucin-like glycopeptide antigen analogues

Florian Karch and Anja Hoffmann-Röder*

Address: Institut für Organische Chemie, Johannes Gutenberg-Universität Mainz, Duesbergweg 10–14, D-55128 Mainz, Germany, Phone: +49-6131-3922417, Fax: +49-6131-3924786

Email: Anja Hoffmann-Röder - hroeder@uni-mainz.de

*Corresponding author

Table of contents

NMR spectra and HPLC chromatogram of compound **2a**	2
NMR spectra of compound **2b**	4
NMR spectra of compound **4**	6
NMR spectra of compound **7**	7
NMR spectra of compound **8**	8

Compound **2a** (300 MHz, CD_3OD): ^1H

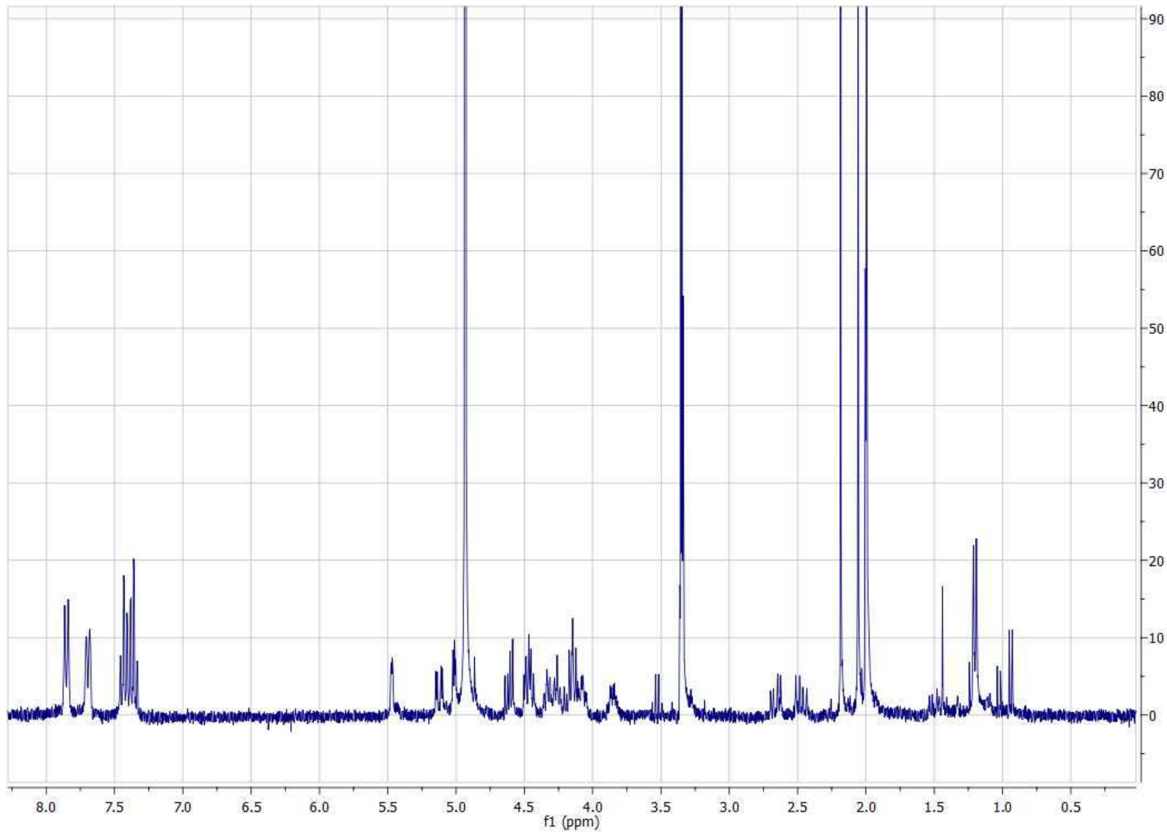

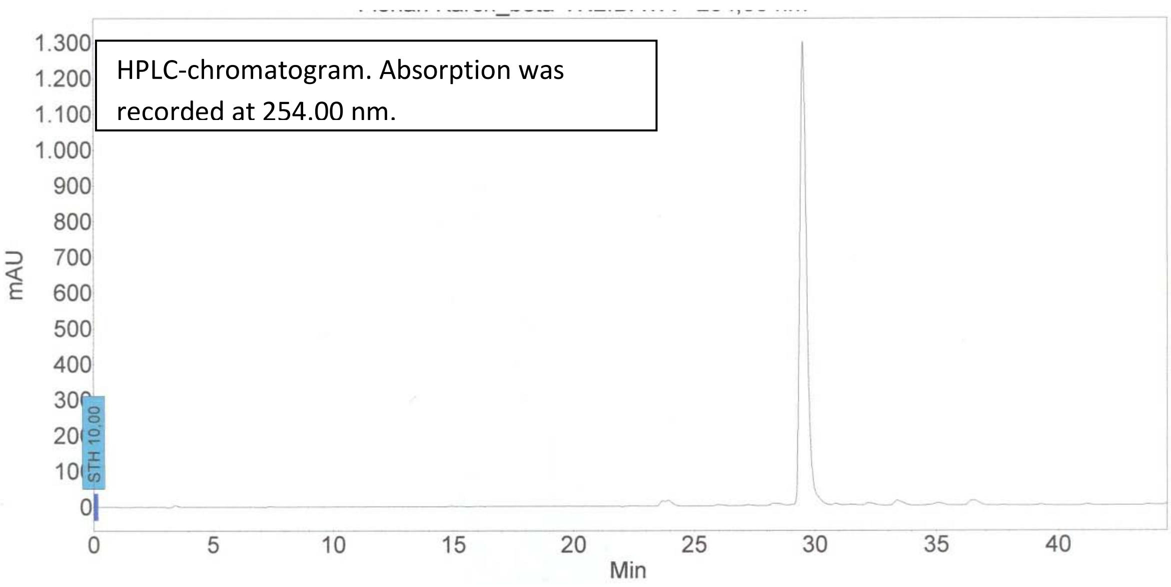

Compound **2a** (400 MHz, CD_3OD): ^{1}H, ^{13}C, H,H-COSY

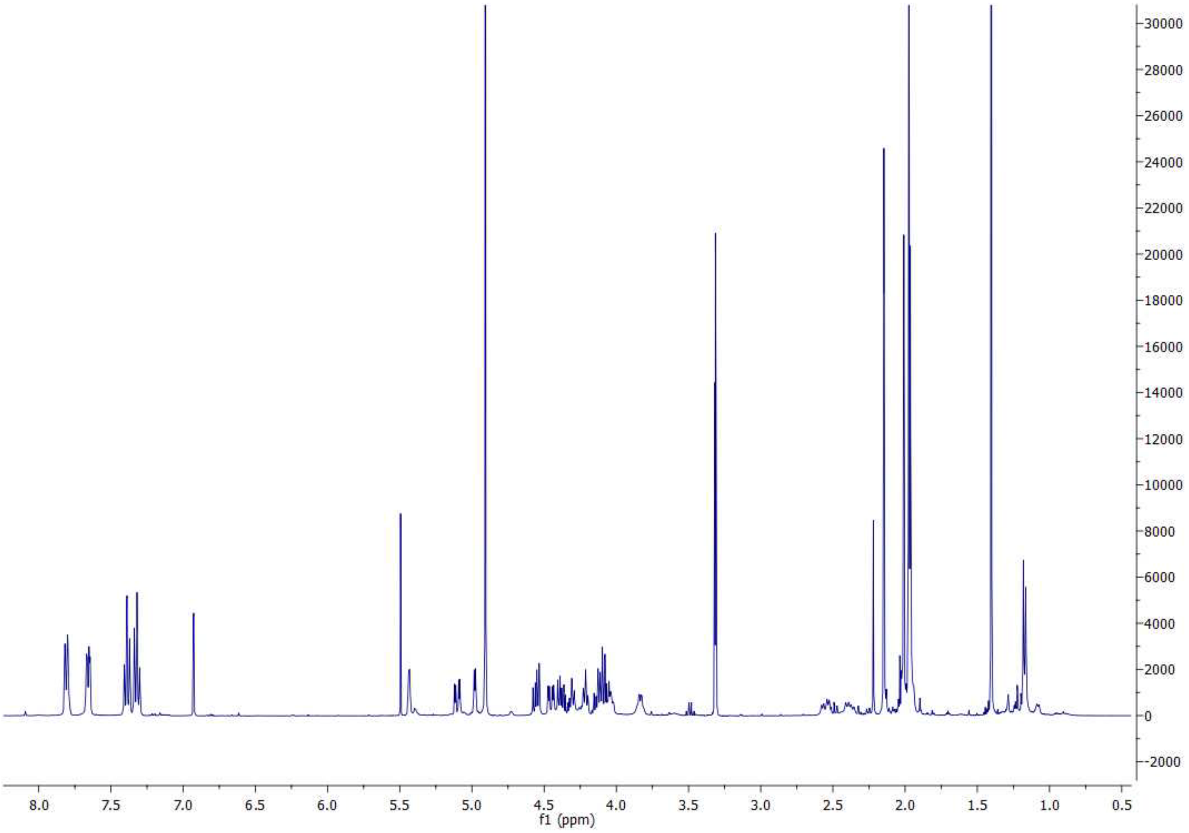

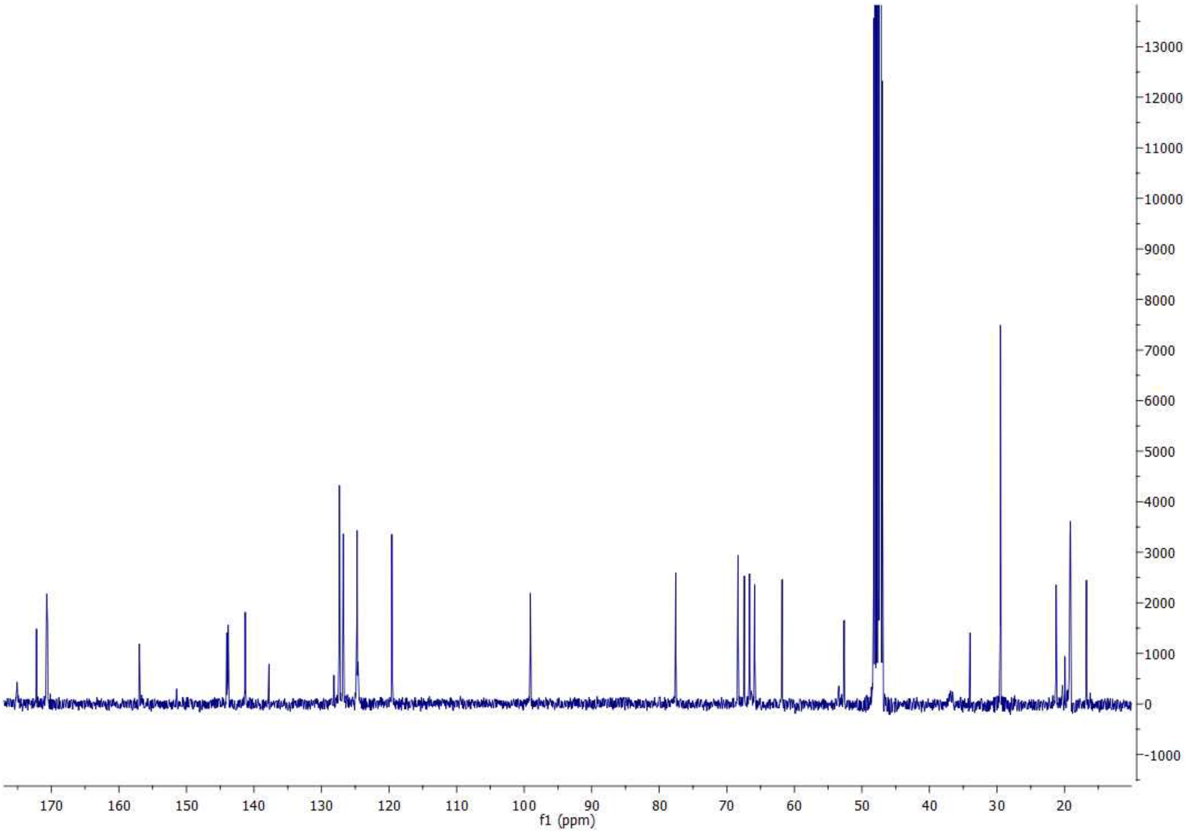

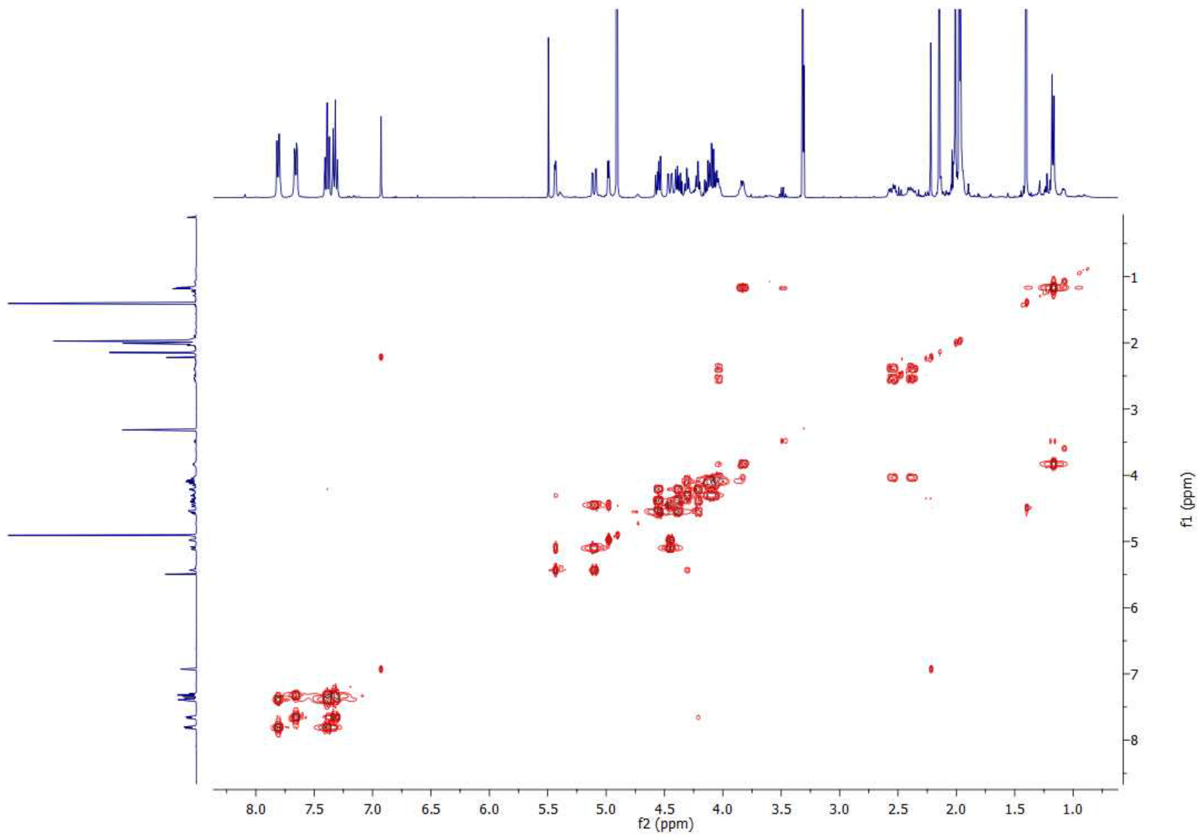

Compound **2b** (400 MHz, CD_3OD): ^1H, ^{13}C, H,H-COSY

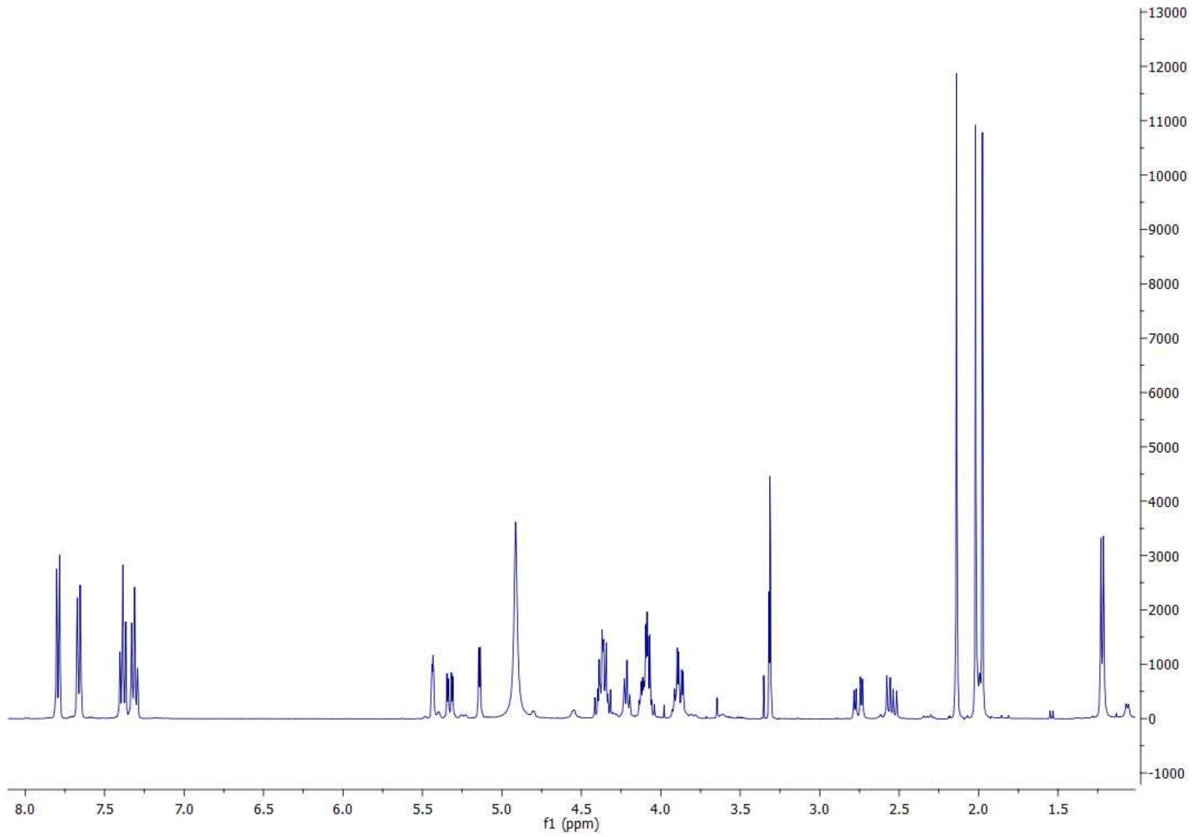

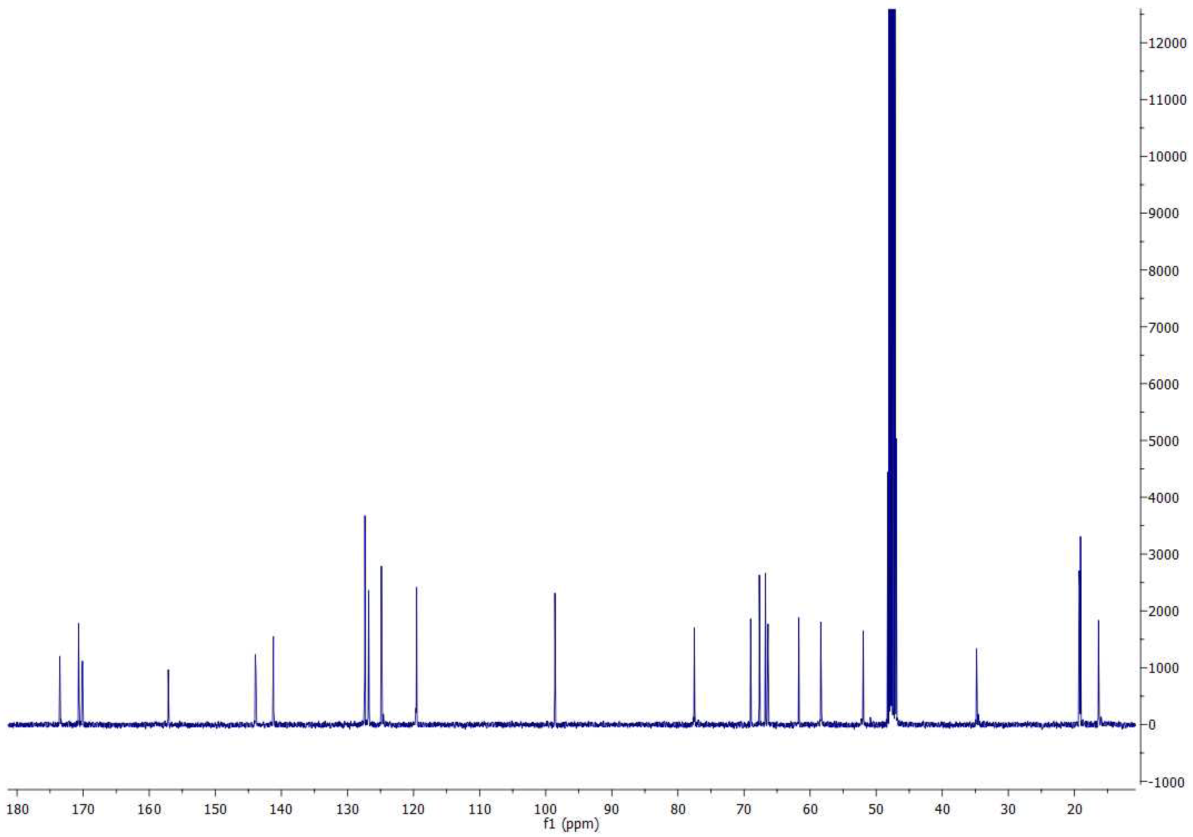
f1 (ppm)

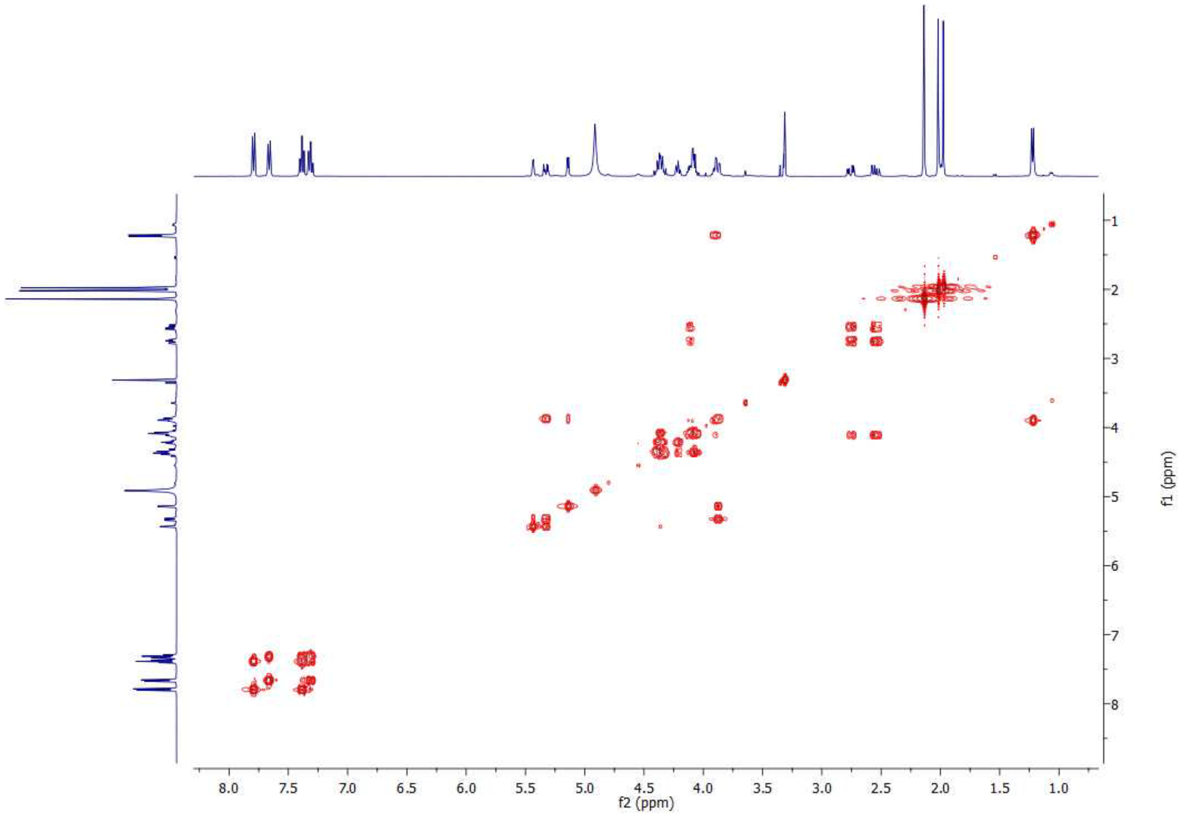
f2 (ppm)
f1 (ppm)

Compound **4** (400 MHz, CD_3OD): ^{1}H, ^{13}C, H,H-COSY

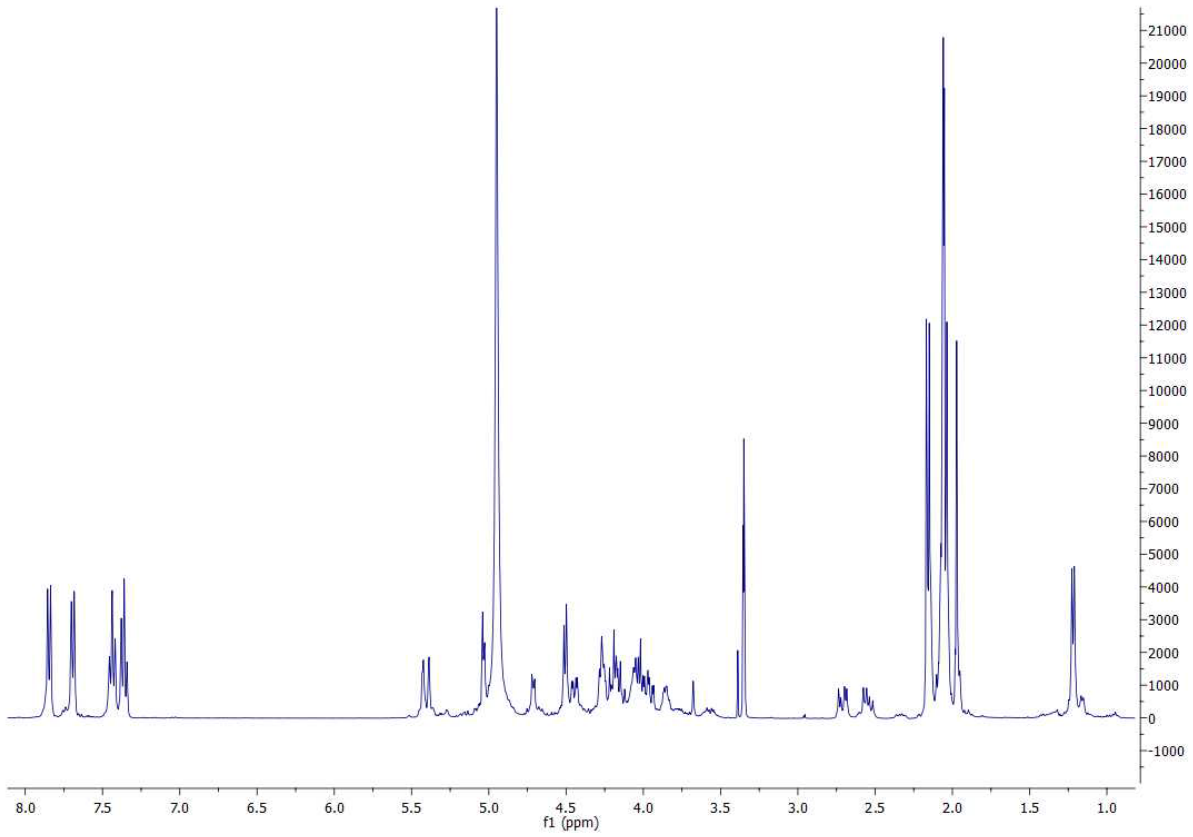

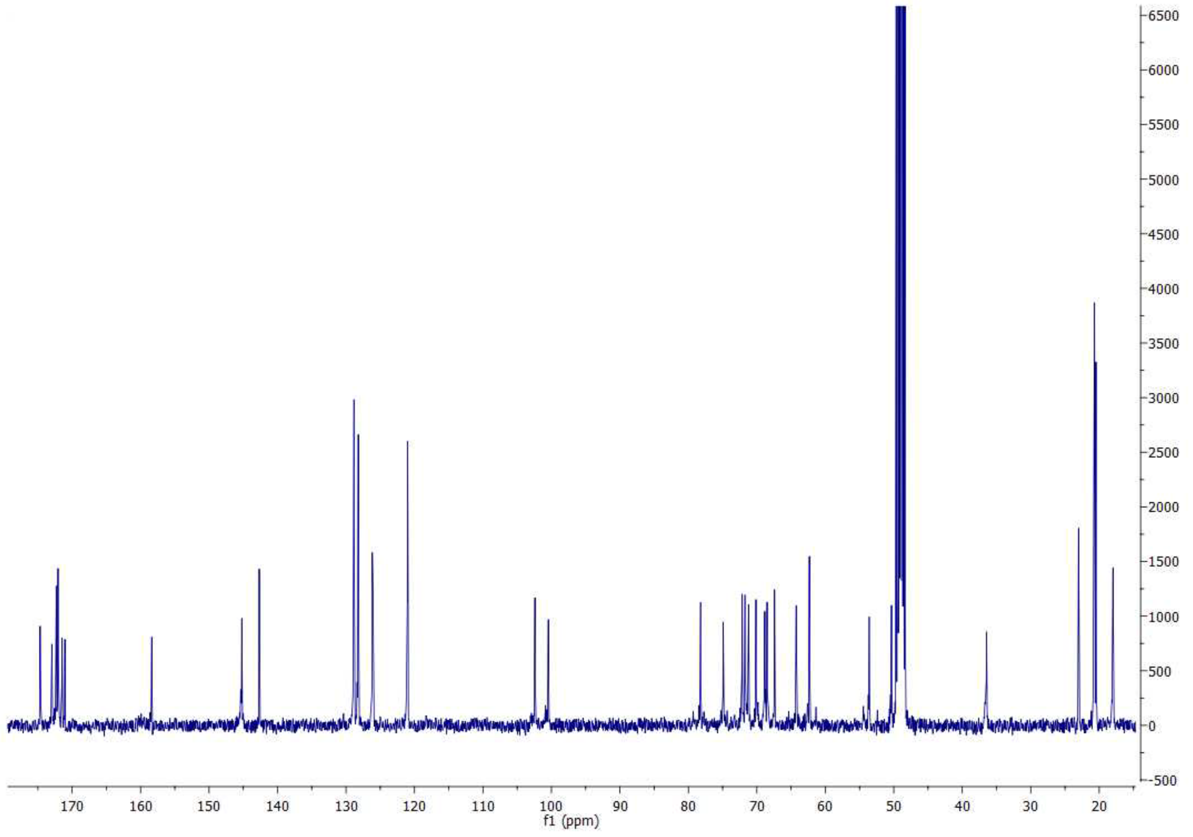

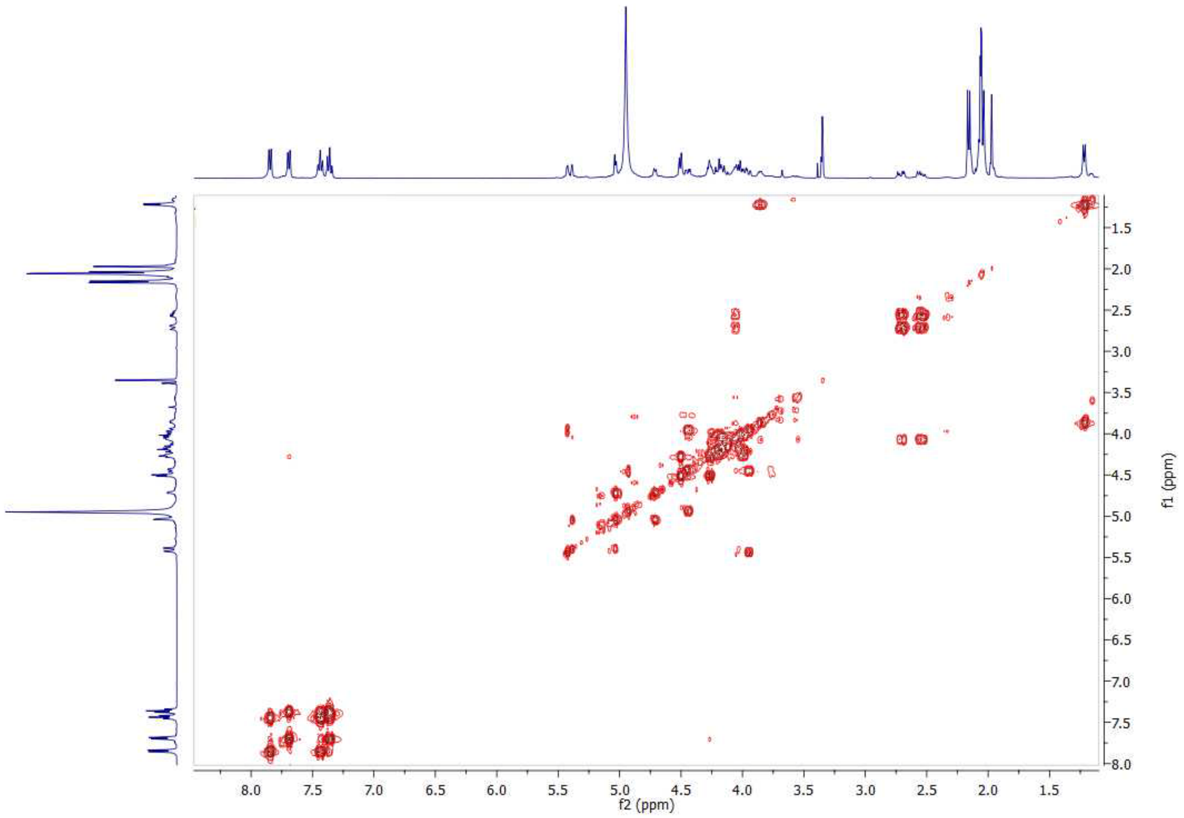

Compound **7** (400 MHz, D_2O): ^{1}H, H,H-COSY

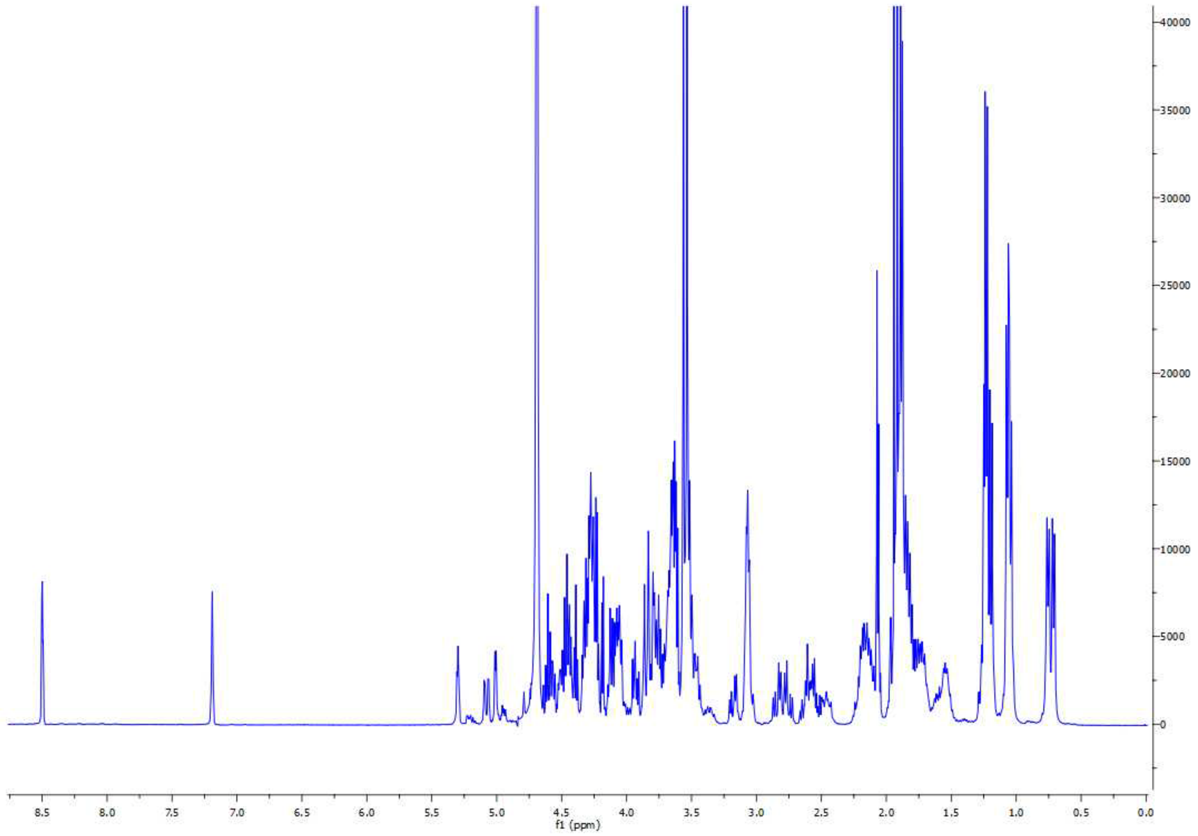

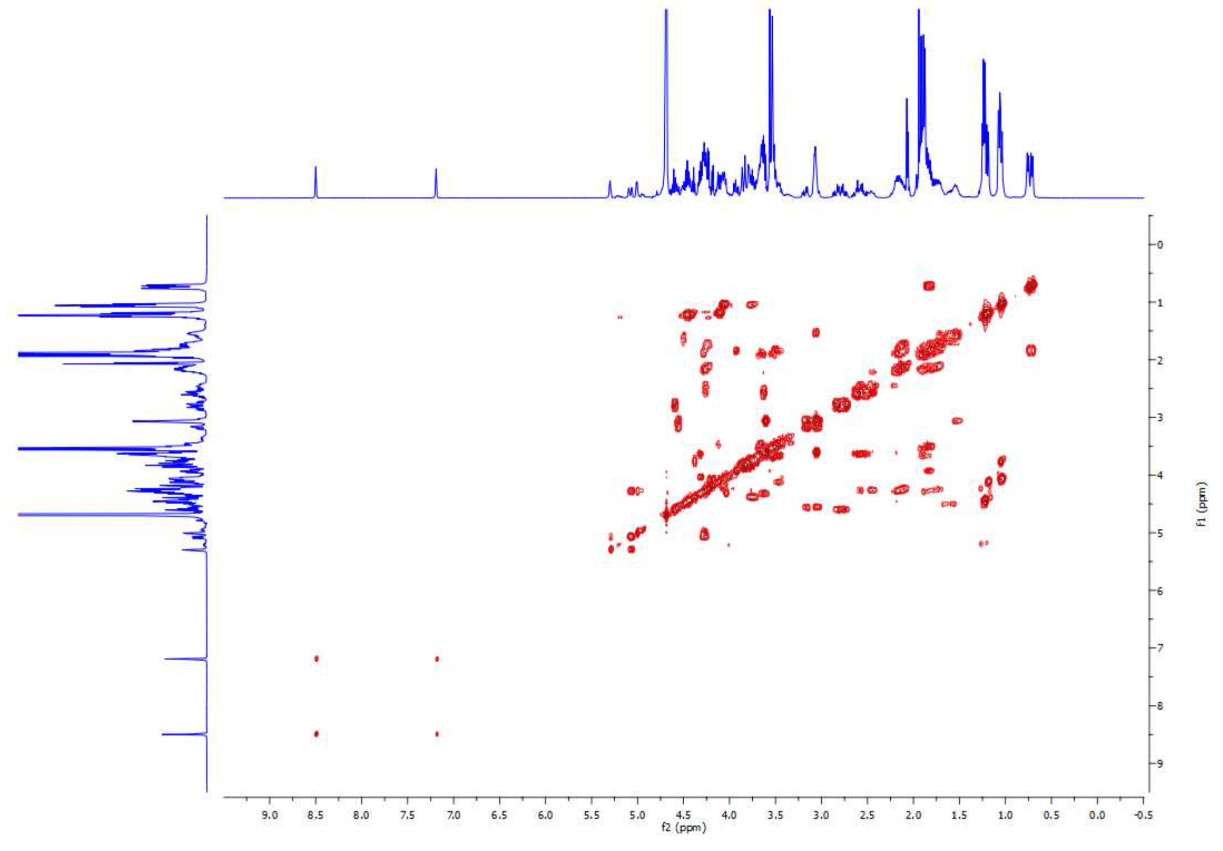

Compound **8** (400 MHz, D_2O): ^{1}H, H,H-COSY

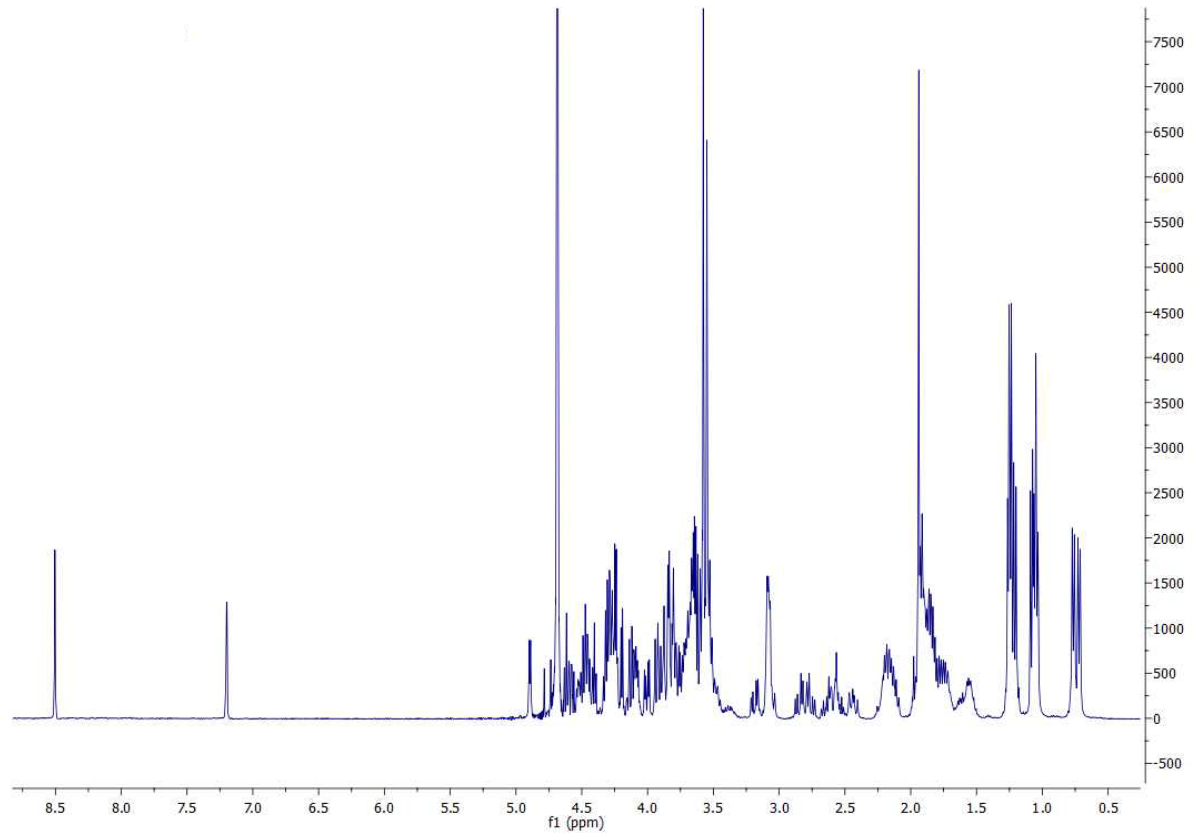

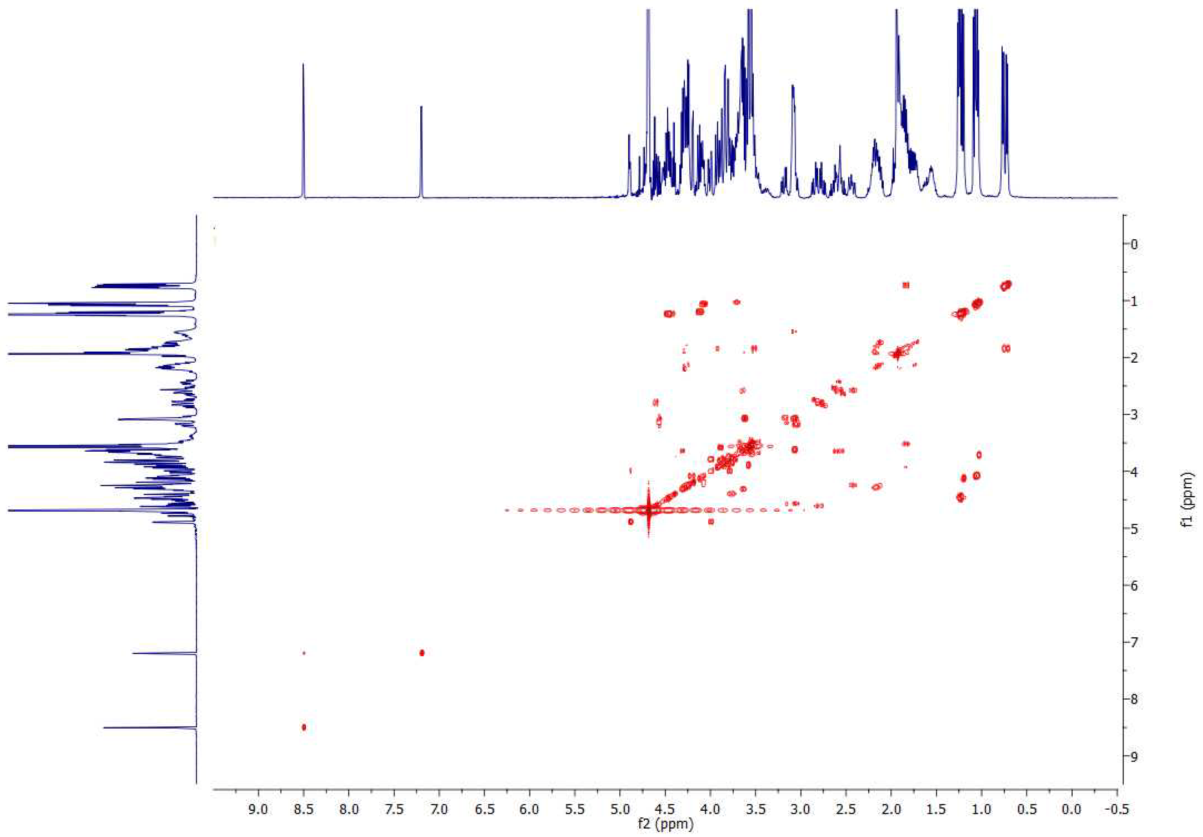
9.0
8.5
8.0
7.5
7.0
6.5
6.0
5.5
5.0
4.5
4.0
3.5
3.0
2.5
2.0
1.5
1.0
0.5
0.0
-0.5
f2 (ppm)
0
1
2
3
4
5
6
7
8
9
f1 (ppm)
